# Deaths from Chloroform

**Published:** 1869-03

**Authors:** 


### Miscellaneous.

### Deaths from Chloroform.

A case of death by chloroform is reported (*Brit. Med. Journal*, December 19, 1868,) in London. A druggist, who had been in the habit of taking chloroform to relieve pain in the face, was found dead in the evening with a handkerchief in his right hand and an empty phial, which had contained chloroform.

Another case of similar kind is recorded by Dr. W. B. Slayter, in the *Provincial Medical Journal* (November, 1868.) A delicate man was found dead in his bed, with a bottle of chloroform lying beside him. From the evidence at the inquest, it appeared that he had been in the habit of inhaling chloroform from time to time to relieve the paroxysms of asthma. On the night of his death he took a little over an ounce.

The same physician records, in the same journal, still another case, which occurred in the Provincial and City Hospital. A man, aged forty, was placed under the influence of chloroform for amputation of thigh owing to inflammation of knee-joint. Chloroform was administered in the usual way on a towel. In a few minutes the patient was fully under its influence, breathing good, pulse strong. The limb was then amputated about the middle third of the thigh, the arteries were tied without delay, and about the usual quantity of blood was lost. Immediately the leg was off, the chloroform was discontinued; at that time the patient was breathing naturally, and the pulse was very good. About three or four minutes after this, the teeth became firmly clinched, respirations stertorous and gasping, pulse very small, and skin covered with a clammy perspiration. The jaws were immediately forced open and the tongue drawn forward; artificial respiration, stimulants, and other remedies were applied, but in vain. The patient died about ten minutes after the first alarming symptoms set in. On examining the diseased joint the synovial membrane was found to be converted into a gelatinous mass; the cartilage covering the inner condyle was perfectly sound, that covering the outer condyle, the heads of the tibia and fibula, was completely destroyed and the bones roughened. *Post mortem* examination about thirty hours after death. The heart substance, valves and aorta, were perfectly healthy, cavities quite empty. The lungs, stomach, spleen, intestines, and kidneys were all healthy, but quite pale from want of blood. The brain was quite pale, and its blood vessels empty. —*Medical News and Library.*

